# Ulnar head replacement or head resection in patients with distal radioulnar arthritis: a prospective cohort study of clinical and patient-reported outcomes up to 2 years after surgery

**DOI:** 10.2340/17453674.2025.44595

**Published:** 2025-09-17

**Authors:** Maria MOLONEY, Sara LARSSON, Elisabeth BROGREN

**Affiliations:** 1 Department of Plastic Surgery, Hand Surgery, and Burns, Linköping University; 2 Department of Clinical and Experimental Medicine, Linköping University; 3 Department of Hand Surgery, Skåne University Hospital, Lund University; 4 Department of Translational Medicine, Malmö, Lund University, Sweden

## Abstract

**Background and purpose:**

Traditional surgery for arthritis of the distal radioulnar joint (DRUJ), which typically involves resecting the ulnar head, is being increasingly challenged by newer techniques, such as prosthetic ulnar head replacement. The aim of our prospective cohort study was to investigate the clinical and patient-reported functional results, up to 2 years postoperatively, among patients with DRUJ arthritis treated with ulnar head replacement or resection.

**Methods:**

40 patients were included and underwent either ulnar head replacement (n = 22) or ulnar head resections (n = 18), due to DRUJ pathology between 2015 and 2020. Patients were followed up at 3, 6, 12, and 24 months postoperatively by the means of Patient-Rated Wrist Evaluation (PRWE) (primary outcome), and Disability of the Arm, Shoulder and Hand (DASH) questionnaires, pain, range of forearm rotation, and grip strength (secondary outcomes). Postoperative complications were recorded. 19 and 16 patients, respectively, responded at the 24-months follow-up. Female sex and inflammatory arthritis were more common in the resection group. General linear regression analyses adjusting for diagnosis and baseline PRWE score were performed for our primary outcome.

**Results:**

The median and interquartile range (IQR) improvement in PRWE from baseline to 24 months was 69 (IQR 49–87) to 27 (IQR 6–48) in the replacement group and 60 (IQR 50–86) to 23 (IQR 5–44) in the resection group, indicating that both groups improved from baseline. There were no differences in unadjusted estimates at any time point. The adjusted means in PRWE at 24 months were 35 and 26 points in the replacement and resection groups, respectively, corresponding to a statistically insignificant mean difference of 8.6 (95% confidence interval –11.7 to 28.2). We found no statistically significant group differences in any of the secondary outcomes at any time point. Postoperative complications affected 6 patients with ulnar head replacement, whereas none were reported for patients with ulnar head resection.

**Conclusion:**

We found that the outcome after ulnar head replacement is not superior to ulnar head resection in the short term.

Arthritis of the distal radioulnar joint (DRUJ) can be a painful condition that is difficult to treat regardless of being caused by inflammatory disease or by osteoarthritis (OA). Traditional surgery with resection of the ulnar head, promoted by Darrach in 1912, is still frequently used for treating DRUJ pathology but can only establish forearm rotation at the expense of forearm stability. The reported outcomes after ulnar head resection are mostly based on retrospective case series showing generally favorable results regarding patient-reported function, pain, and range of motion in both the short and long term [1-4]. During the 1980s and ’90s, several authors reported problems with the Darrach procedure, including instability of the distal ulna, ulnar carpal translocation, decreased grip strength, and pain [5-7]. Awareness of the biomechanical significance of an intact DRUJ in terms of load transmission and restoration of the normal axis of forearm rotation led to increased interest in prosthetic replacement of the ulnar head [8-10]. The clinical results have shown encouraging results with improved function, effective pain relief, and preserved forearm rotation, which has contributed to the growing popularity of ulnar head replacement [11-14]. However, the early reports linking poor outcomes to ulnar head resection often involved excessive distal resection of the ulna and poor reconstruction of the soft tissues, suggesting the procedure’s reputation may be undeserved [3]. Recent studies have shown that even if convergent instability was a common radiological finding, the phenomenon did not correspond to poor functional outcome [2,4]. The biomechanical performance of ulnar head resection in comparison with ulnar head replacement was evaluated, showing improved forearm stability with the latter [15], but, surprisingly, comparative clinical studies are still lacking [16]. As implementation of any new surgical method should rely on evaluation against the current gold standard, we performed the current study.

The aim of our prospective cohort study was to investigate the clinical and patient-reported functional results, up to 2 years postoperatively, among patients with DRUJ pathology surgically treated with ulnar head replacement or ulnar head resection. The primary outcome was the Patient-Rated Wrist Evaluation (PRWE) score. Disability of the Arm, Shoulder and Hand (DASH) score, pain, range of forearm rotation, and grip strength were secondary outcomes.

## Methods

### Study design and population

This is a single-center, prospective, longitudinal cohort study. All eligible patients who underwent ulnar head replacement or ulnar head resection at the Department of Hand Surgery at Malmö University Hospital, Sweden, between March 2015 and February 2020 were enrolled. The department is the main facility to which patients with wrist arthritis are referred and the only center in the area that performs ulnar head replacements (population during the study period approximately 1.9 million inhabitants). Patients were identified preoperatively at the outpatient clinic and enrolled after informed oral and written consent. Inclusion criteria were age above 18 and DRUJ arthritis due to an inflammatory (e.g., rheumatoid or psoriatic arthritis [IA]) or a noninflammatory (e.g., idiopathic or posttraumatic osteoarthritis [OA]) arthritis. We excluded patients with severe cognitive disorders, drug or alcohol abuse, or language difficulties who were unable to complete questionnaires from inclusion in the study. The study was reported according to STROBE guidelines.

### Surgical methods

Patients were eligible for both procedures, and the hand surgeon’s choice of surgical method was based on patient medical history, clinical and radiological evaluation of the wrist, and the patients’ own requests. Additional surgery was performed at the same time to address the radiocarpal/intercarpal joints or tendon transfers if needed. All patients who underwent an ulnar head replacement received the Herbert ulnar head prosthesis (KLS Martin, Tuttlingen, Germany) [17]. The Herbert ulnar head prosthesis, released on the market in 1995, consists of a non-cemented titanium stem with a ceramic head.

The surgery for ulnar head replacement was performed as described by Herbert and Schoonhoven [17]. The distal ulna is accessed through a dorsal incision, and the fifth compartment is identified and incised. An ulnar based capsulo-retinacular flap including the extensor carpi ulnaris tendon is raised, which after insertion of the prosthesis is attached to the dorsal rim of the sigmoid notch through osteosutures or suture anchors to provide stability to the DRUJ. Using a surgical saw, the ulnar osteotomy is performed as determined by the resection guide. The ulnar head is carefully dissected and removed. The ulnar shaft is then reamed until appropriate diameter is achieved and a trial prosthesis is used to ensure the fit before the definitive prosthesis is inserted.

For patients who underwent ulnar head resection (i.e., the Darrach procedure), access to the DRUJ was performed similar to that for ulnar head replacement surgeries, although a longitudinal incision to the dorsal capsule was performed, and the capsule was sutured rather than anchored after resection. The ulna was transected at the proximal level of the sigmoid notch of the radius, and the ulnar head was completely resected. Care was taken to smooth any sharp edges of the stump. Finally, the stump was stabilized using a volar capsule flap attached to the dorsal ulna through osteosutures and the dorsal capsule was repaired with non-resorbable sutures.

### Clinical and patient-reported outcomes

Patients were followed for 2 years after surgery with evaluations at baseline (i.e., preoperatively), and at 3, 6, 12, and 24 months postoperatively. Patients were asked to complete the validated Swedish versions of 2 Patient Reported Outcome Measures (PROMs) on all occasions: the Patient Rated Wrist Evaluation (PRWE) and Disability of the Arm, Shoulder and Hand (DASH) [18,19]. Both questionnaires score disability on a scale from 0 (no disability) to 100 (severe disability) [18,19]. PRWE, which is a wrist-specific PROM that measures perceived disability and pain in the affected wrist joint, was our primary outcome [19]. DASH measures self-reported upper extremity physical function and symptoms, taking the whole upper extremity into account [18]. Both PRWE and DASH have shown excellent psychometric properties in wrist osteoarthritis [20]. Patients were also asked to rate the severity of their wrist pain at rest, on motion, and on loading the wrist, using a visual analog scale (VAS) from 0 (no pain) to 100 (severe pain) at baseline and on all follow-up occasions [21].

The range of forearm rotation regarding pronation and supination was measured in the operated hand with a goniometer. Grip strength of both hands was measured with a dynamometer (Saehan Hand Dynamometer; Saehan Corp, Gyeongsangnam-do, Republic of Korea). 3 trials were recorded for each hand and the mean value was calculated. Grip strength of the operated hand was presented as percentages of the grip strength in the contralateral hand. The measurements were made with the same equipment and performed by the same physiotherapist (SL) on all occasions.

### Radiographic outcome

**Figure 1:**
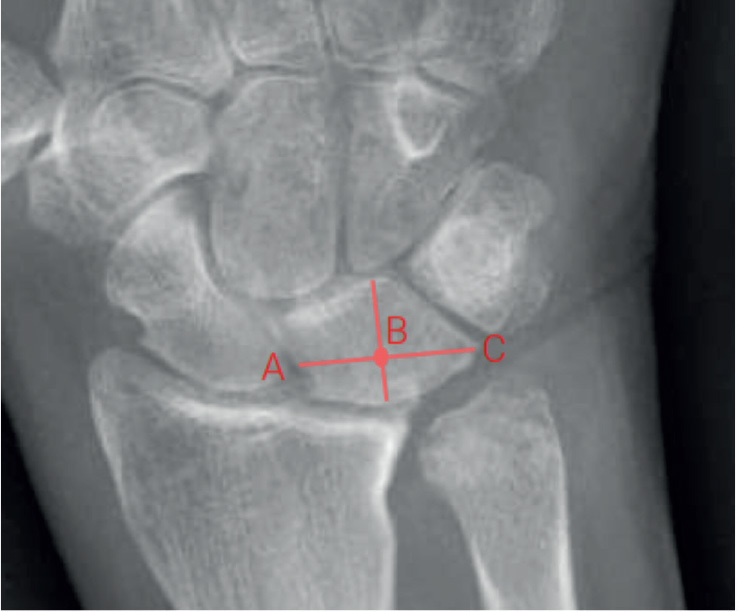
Ulnar carpal translation was calculated by dividing the distance B–C by the total lunate width A–C.

Ulnar carpal translation was measured in posteroanterior radiographs with the hand in a neutral position, according to the method originally described by Wollstein et al. [22] (Figure 1). A lunate uncovering of >50% was considered as ulnar translation [22]. The measurements were performed by MM, who was blinded to patient characteristics and surgical method through anonymized, cropped, and randomized radiographs.

### Complications

Postoperative complications related to the DRUJ surgery were recorded and classified as early (occurring within the first postoperative month) or late (occurring after the first postoperative month) complications.

### Sample size

The sample size was calculated based on previous studies of minimal clinically important differences (MCIDs) of our primary outcome PRWE ranging from 11.5 to 14 [23,24]. To detect a difference of 13 between the mean PRWE in the ulnar head replacement group and the ulnar head resection group, with a standard deviation (SD) of 14, power of 80%, and significance level of 0.05, a minimum of 19 patients in each group was needed.

### Statistics

Statistical calculations were performed using IBM SPSS Statistics data Editor (IBM Corp, Armonk, NY, USA). As the data was not normally distributed according to the Shapiro–Wilks test, we used non-parametric statistics. For categorical values (sex, diagnosis, additional surgery) at baseline, the standardized difference for proportions was calculated to assess the magnitude of the difference between the groups. For all other baseline variables (age, PRWE, DASH, VAS pain, grip strength, and range of forearm rotation) the Mann–Whitney U-test was performed, and the effect sizes (r) were computed to quantify the differences between the groups. Median differences for the primary and all secondary outcomes at 6 and 24 months were calculated from the original data. Corresponding 95% confidence intervals (CI) were estimated using non-parametric bootstrap resampling (1,000 iterations with replacement), based on the percentile method. Separate general linear regression analyses were conducted at 3, 6, 12, and 24 months to compare groups on the primary outcome (PRWE), adjusting for diagnosis (IA vs OA) and baseline PRWE score as a continuous covariate. Equivalence was assessed using a pre-specified margin of ±13 points for PRWE, based on MCID [23,24]. Differences within this range were considered not clinically meaningful. Within-group differences between baseline and 24 months were analyzed with the Wilcoxon signed rank-test.

Data completeness varied across time points and outcome measures, mainly due to incomplete questionnaires or patients cancelling their appointments due to the COVID-19 pandemic. Missing values were assumed to be missing at random. For the primary outcome (PRWE) and the outcome with highest missing data (grip strength), we conducted sensitivity analyses to assess the potential impact of missing data at the 6- and 24-month follow-up. In the sensitivity analyses we applied the last observation method, in which the most recent available value for each patient at the 3-month and 12-month follow-up, respectively, was carried forward to replace missing 6- and 24-month values (25).

### Ethics, data sharing plan, funding, use of AI, and disclosures

This study was approved by the Lund University Ethical Board, Sweden (No. 2015/121). No data sharing plan was implemented for this study, as consent for data sharing was not obtained from the included patients at the time of recruitment. The study was supported by grants from Kockska Stiftelsen. The funders had no role in study design, data collection, analysis, or manuscript preparation. No AI tools were used. The authors have no disclosures to report; complete disclosure of interest forms according to ICMJE are available on the article page, doi: 10.2340/17453674.2025.44595

## Results

### Patients and baseline characteristics

**Figure 2:**
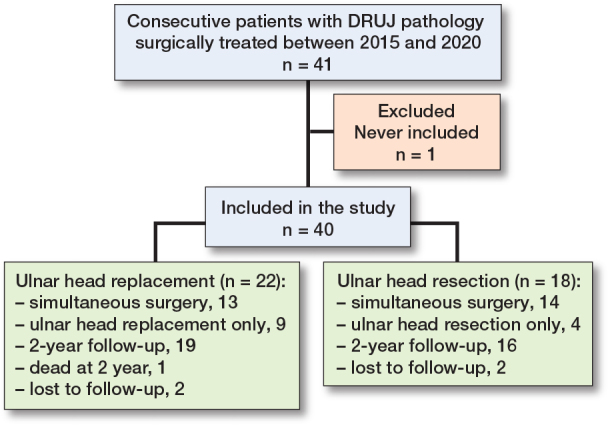
Patient flowchart.

41 patients, of whom 22 underwent ulnar head replacement and 19 ulnar head resections, were eligible. One patient with ulnar head resection was never included, and thus a total of 40 patients with a median age of 66 (interquartile range [IQR] 52–75) years were included in the study (22 ulnar head replacements and 18 ulnar head resections). One patient with ulnar head replacement died between the 12- and 24-month follow-up (Figure 2).

Female sex and a diagnosis of IA was more common among patients who received an ulnar head resection (Table 1). The indication for surgery was DRUJ pain in the majority of patients, and extensor tendon rupture caused by sharp edges of the ulnar head was diagnosed in 6 of 22 patients with ulnar head replacement and in 8 of 18 patients with ulnar head resection (Table 1). Additional surgery with total wrist fusion in combination with tendon transfer was more common among patients who received ulnar head resection compared with patients who received ulnar head replacement, but no other differences in type or frequency of additional surgery was found between the groups (Table 1). Nor were there any differences in patient-reported pain and function, range of forearm rotation, or grip strength at baseline (Table 1). In 3 of the patients with ulnar head replacement, bone cement was used for fixation of the stem in the distal ulna.

**Table 1:** Baseline characteristics. Continuous data are shown as median (interquartile range) and categorical as numbers

Item	Ulnar head replacement (n = 22)	Ulnar head resection (n = 18)	Stand. diff. ^a^
Age at surgery	63 (50–71)	70 (53–77)	0.16
Sex (male/female)	14/8	3/15	1.09
Diagnosis (IA/OA)	9/13	16/2	1.16
Dominant side (yes/no/unknown)	11/8/3	6/8/4	0.36
Indications for DRUJ surgery			
Pain	16	10	0.37
Pain and extensor tendon rupture	4	7	0.48
Extensor tendon rupture	2	1	0.14
Additional surgery	13	14	0.42
Total wrist fusion	3	4	0.21
Partial wrist fusion	4	0	0.66
Tendon transfer	6	3	0.24
Wrist fusion + tendon transfer	0	5	0.88
Soft tissue surgery	0	2	0.5
Preoperative antibiotics	21	12	0.81
PRWE ^b^	69 (49–87)	60 (50–86)	0.02
DASH ^b^	48 (30–63)	52 (28–60)	0.04
VAS pain at rest ^c^	43 (14–68)	34 (10–69)	0.07
VAS pain on motion ^c^	66 (49–85)	58 (31–90)	0.03
VAS pain on load ^c^	83 (71–95)	76 (55–90)	0.17
Supination (degrees) ^c^	65 (49–70)	65 (51–70)	0
Pronation (degrees) ^c^	70 (65–75)	70 (66–79)	0.02
Grip strength (%) ^d,e^	67 (59–88)	70 (38–84)	0.25

IA = inflammatory arthritis, OA = osteoarthritis, DRUJ = distal radioulnar joint, PRWE = Patient-Reported Wrist Evaluation, DASH = Disability of the Arm, Shoulder and Hand, VAS = Visual Analogue Scale.

a Standardized differences are presented for proportions and effect sizes (r) for continuous variables.

b Missing 4 values (1 ulnar head replacement, 3 ulnar head resection).

c Missing 2 values (2 ulnar head resection).

d Missing 7 values (3 ulnar head replacement, 4 ulnar head resection).

e Grip strength presented as the percentage of the contralateral wrist.

### Patient-reported outcomes

At baseline, 36 patients had valid data for PRWE, DASH, and range of forearm rotation, 38 patients for VAS pain and 33 for grip strength. At 3 months, valid data was available for 35 patients for PRWE and DASH, 36 for VAS pain and range of forearm rotation, and 33 for grip strength. At 6 months, valid data was available for 37 patients with PRWE, DASH, and VAS pain, 36 for range of forearm rotation, and 34 for grip strength. At 12 months, 35 patients had PRWE, DASH, VAS pain, and range of forearm rotation data, and 33 had grip strength. At 24 months, 35 patients had PRWE and VAS pain, 34 had DASH scores and range of forearm rotation, and 32 had grip strength.

**Figure 3:**
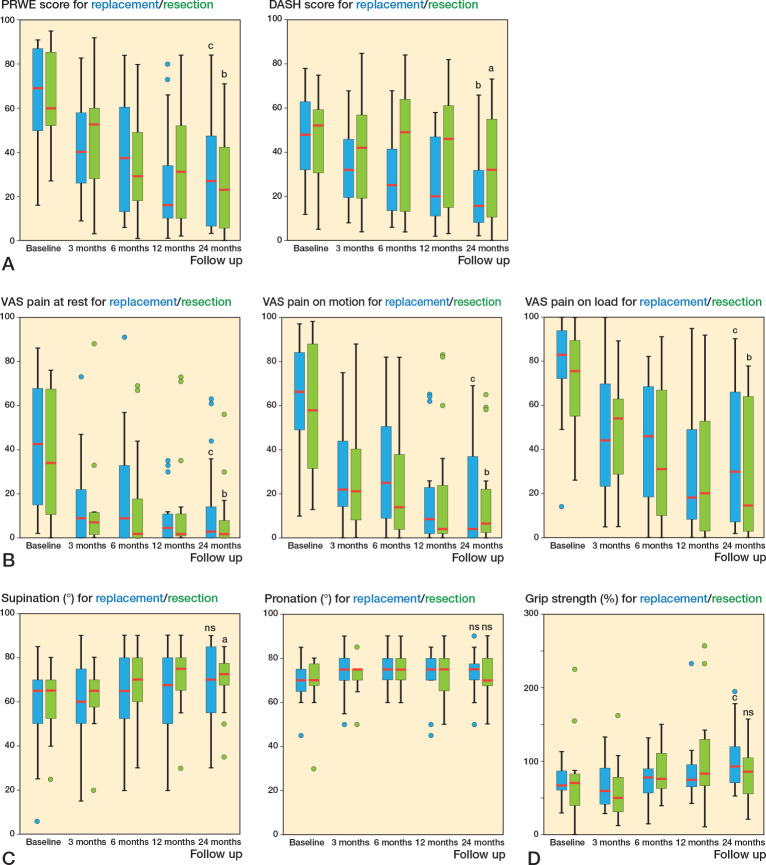
Box plots showing the median (red line), interquartile range (box), values within 1.5 times the interquartile range width (whiskers), and outlier values (dots outside whiskers) for (A) the PRWE and DASH, (B) VAS pain at rest, VAS pain on motion without loadin, and VAS pain on loading, (c) forearm pronation and supination, and (D) grip strength for the ulnar head replacement and ulnar head resection groups at baseline and at 3, 6, 12, and 24 months postoperatively. Within-group differences from baseline to 24 months were measured with the Wilcoxon signed rank-test. ns = non-significant, P value ^a^ < 0.05, ^b^ < 0.01, ^c^ < 0.001.

There were no differences in patient-reported disability or pain between the ulnar head replacement and ulnar head resection groups at any follow-up time point (Figure 3). Although the variability was large between patients, both groups improved in PRWE, DASH, and VAS pain scores between baseline and 24 months postoperatively (Figure 3). The unadjusted median differences in PROMs at 6 and 24 months, and their CIs, are presented in Table 2.

**Table 2:** Unadjusted estimates for primary and secondary outcomes at 6 and 24 months. Values are median (interquartile range) and median difference with (95% confidence intervals)

Outcome	Ulnar head replacement	Ulnar head resection	Median difference
PRWE			
6 months	38 (13–61)	29 (14–53)	9 (–19 to 30)
24 months	27 (6–48)	23 (5–44)	4 (–19 to 34)
DASH			
6 months	25 (13–42)	49 (13–67)	–24 (–33 to 12)
24 months	16 (8–36)	32 (10–56)	–16 (–35 to 1)
VAS pain at rest			
6 months	9 (0–34)	2 (0–23)	7 (–2 to 16)
24 months	3 (0–15)	2 (0–10)	1 (–3 to 7)
VAS pain on motion			
6 months	25 (8–51)	14 (4–44)	11 (–17 to 29)
24 months	4 (0–38)	7 (2–24)	–3 (–11 to 14)
VAS pain on load			
6 months	46 (16–70)	31 (9–68)	15 (–20 to 38)
24 months	30 (6–71)	15 (3–69)	15 (–13 to 39)
Supination (°)			
6 months	65 (50–80)	70 (60–80)	–5 (–23 to 10)
24 months	70 (55–90)	73 (66–79)	–3 (–20 to 10)
Pronation (°)			
6 months	75 (70–80)	75 (70–80)	0 (–5 to 5)
24 months	75 (70–80)	70 (66–83)	5 (–5 to 8)
Grip strength (%) ^a^			
6 months	78 (55–92)	91 (62–114)	–13 (–49 to 10)
24 months	93 (70–122)	93 (47–107)	0 (–27 to 50)

a Grip strength presented as the percentage of the contralateral wrist.

For abbreviations, see Table 1.

The adjusted mean differences in PRWE scores at 3, 6, 12, and 24 months showed consistent results after controlling for baseline PRWE values and diagnosis (Table 3). Although the adjusted mean differences did not reach an MCID of 13 in PRWE at any time point, the confidence intervals exceeded the equivalence margin, suggesting that clinically meaningful difference between the groups could not be fully ruled out. Sensitivity analyses addressing missing data did not alter the findings for PRWE at 6 and 24 months in either the unadjusted or adjusted analyses (data not shown).

**Table 3:** Adjusted ^a^ means and differences with 95% confidence intervals (CI) for the primary outcome at 3, 6, 12, and 24 months

PRWE at	Ulnar head replacement	Ulnar head resection	Adjusted mean difference (CI)
3 months	43.6	46.3	–2.7 (–18.2 to 12.8)
6 months	41.4	36.6	4.8 (–15.1 to 24.7)
12 months	30.0	29.4	0.6 (–20.1 to 21.3)
24 months	34.6	26.0	8.6 (–11.7 to 28.8)

a Adjusted for diagnosis (inflammatory arthritis/osteoarthritis) and baseline value for PRWE.

For abbreviations, see Table 1.

### Clinical outcomes

Range of forearm pronation was near normal at baseline (26) and not affected by surgery. There was no difference in forearm rotation between patients with ulnar head replacement and patients with ulnar head resection at any follow-up time point (Table 2 and Figure 3). The variability in forearm pronation between patients was smaller compared with the measurements of forearm supination, especially among patients with ulnar head replacement (Figure 3). This may explain why patients who received ulnar head resection had a statistically significant within-group improvement in supination from baseline (median 65° [IQR 51–70]) to 24 months postoperatively (median 73° [IQR 66–79]), P = 0.03, whereas patients with ulnar head replacement did not improve their forearm supination at group level (baseline median 65° [IQR 49–70] to 70° [IQR 55–90] at 24 months, P = 0.07).

Grip strength (% of contralateral hand) showed, among patients with ulnar head replacement, improvement from median 67% (IQR 59–88) at baseline to 93% (IQR 70–122) at 24 months (P < 0.001) (Figure 3). The corresponding values for grip strength among patients with ulnar head resection (70% [IQR 38–84] at baseline and 93% [IQR 47–107] at 24 months), however, did not reach statistical significance (P = 0.7) (Figure 3). The largest proportion of missing data was observed for grip strength, but the unadjusted sensitivity analyses at 6 and 24 months did not alter the results (data not shown).

### Radiographic outcome

Ulnar carpal translation was measured in 30 patients (18 ulnar head replacement and 12 ulnar head resection) at baseline and in 14 patients (10 ulnar head replacement and 4 ulnar head resection) at 24 months. It was impossible to measure ulnar carpal translation in 10 patients at baseline (1 patient had no preoperative radiographs and 9 patients had previous wrist surgery or the carpus was too degenerated to measure ulnar carpal translation). At 24 months, it was impossible to measure ulnar carpal translation in 26 patients (in 4 patients due to missing radiographs and in 22 patients due to the type of wrist surgery).

At baseline, 13 of 18 of patients with ulnar head replacement and 10 of 12 of patients with ulnar head resection had ulnar carpal translation (> 50% of lunate uncovering). The corresponding values at 24 months were 6 of 10 and 2 of 4, respectively.

### Complications

During the first 24 months after surgery, 6 of the 22 patients with ulnar head replacement had postoperative complications, whereas no complications were reported for patients with ulnar head resection (Table 4).

**Table 4:** Postoperative complications during the first 2 years after surgery

Complications ^a^	Ulnar head replacement	n	Ulnar head resection	n
Early	Carpal tunnel syndrome 2 days after surgery, treated with carpal tunnel release. Perioperative fracture of ulnar shaft, treated with cerclage wire.	1 2	None	0
Late	Subluxation, patient declined revision. Deep infection, revision after 6 months. Unclear pain, converted after 6 months to ulnar head resection.	1 1 1	None	0

a Early complications occurred within the first month after surgery, and late complications occurred after the first month.

## Discussion

The aim of our prospective cohort study was to investigate the clinical and patient-reported functional results, up to 2 years postoperatively, among patients with DRUJ pathology surgically treated with ulnar head replacement or ulnar head resection. The primary outcome was the PRWE score, and DASH score, pain, range of forearm rotation, and grip strength were secondary outcomes.

We found that both ulnar head replacement and ulnar head resection showed improvement from baseline, but there was no difference between the 2 procedures in terms of patient-reported outcome or pain during the first 2 years after surgery. Notably, 27% of patients who received ulnar head replacement suffered from postoperative complications, compared with none of the patients in the ulnar head resection group. Similar high complication rates (28%) were reported in a previous systematic review of 14 studies with a total of 335 implanted ulnar head replacements [11].

In the present cohort, DRUJ surgery was often performed in conjunction with other procedures, such as partial or total wrist fusion and/or reconstruction of extensor tendon function, complicating the evaluation of results. Additional surgery is frequently reported in previous studies of both ulnar head replacement and ulnar head resection, and addressing concomitant wrist and soft tissue related problems at the same time as treating the arthritic ulnar head could be considered common clinical practice [4,27-29]. Thus, the improvement in PRWE, DASH, and VAS pain scores in our material, as well as in that of others, is probably not attributed to the DRUJ surgery alone.

One of the strongest arguments for replacing the arthritic ulnar head with an implant, rather than choosing simple ulnar head resection, is that the integrity of the DRUJ is necessary for load transmission and stability [8,10]. However, we found no difference in grip strength or VAS pain scores on load between patients who received ulnar head replacement and those who received ulnar head resection at any follow-up time point in our study. The relative grip strength showed a within-group improvement from baseline to 2 years after surgery among patients with ulnar head replacement, but not among patients with ulnar head resection. These findings should, however, be interpreted with caution, as a greater proportion of patients who received ulnar head resection had inflammatory arthritis, suggesting that coexisting pathology such as finger deformities or subluxations may have prevented these patients from improving their grip strength significantly. Moreover, grip strength had the highest proportion of missing data. Although sensitivity analyses yielded consistent results, the ability to draw firm conclusions from these findings is limited.

We found that forearm rotation remained unchanged overall after surgery compared with preoperatively, except for a slight within-group improvement in supination for patients with ulnar head resection. However, the clinical relevance of a median improvement of 8° in supination is questionable.

### Limitations

As the groups were not randomized, there is a risk of selection bias. Due to the pragmatic study design, treatment choice was influenced by patient factors, which is reflected in the greater number of patients with inflammatory arthritis and female sex in the ulnar head resection group compared with the ulnar head replacement group. However, the type of diagnosis was adjusted for in the general linear regression. As mentioned earlier, the majority of patients had additional surgery in conjunction with the DRUJ procedure, which affects the interpretation of the results. This is a common phenomenon in DRUJ surgery that has also been reported by others [4,27-29]. The number of included patients was small, and the study was performed in a single-center setting, although this allowed us to control for missing subjects. However, there is a risk that our study was underpowered, and in combination with the wide confidence intervals this means that we cannot fully rule out a clinically meaningful difference between the groups. Replication of our findings in larger and more homogeneous cohorts is warranted before definitive conclusions can be drawn.

We did not evaluate DRUJ instability with dynamic evaluation methods but focused on using validated PROMs and common clinical measures. The strengths of the study include its longitudinal prospective design and the fact that the same physiotherapist performed all measurements.

Most patients in our cohort had ulnar carpal translocation at baseline, and many had undergone additional surgeries such as carpal arthrodesis. As a result, assessing progressive ulnar translocation of the carpus between baseline and 24 months became less meaningful due to the confounding effects of these pre-existing conditions and surgical interventions.

### Conclusion

Even if the ulnar head resection group was more fragile, with a higher percentage of inflammatory arthritis suggesting a higher risk of coexisting pathology, the ulnar head replacement group showed similar clinical and patient-reported outcomes during the first 2 years after surgery. The risk of complications in the ulnar head replacement group was higher than in the ulnar head resection group.

*In perspective,* based on our study it seems not to be justified to use ulnar head replacement over ulnar head resection in patients with DRUJ pathology.

## Notes

EB was responsible for both the conceptualization and design of the study. Data was collected by SL and EB. All measurements regarding ROM and grip strength were made by SL. Data was sorted by MM and statistical calculations were made by MM and EB. MM drafted the manuscript. Interpretation of the results were made by all authors, who also contributed to critical revision of the manuscript. All authors read and approved the final manuscript.

Handling co-editors: Jeppe Vejlgaard Rasmussen and Robin Christensen.

*Acta* thanks Inga Besmens and Torben B Hansen for help with peer review of this manuscript.
